# Advanced approach to analyzing calcareous protists for present and past pelagic ecology: Comprehensive analysis of 3D-morphology, stable isotopes, and genes of planktic foraminifers

**DOI:** 10.1371/journal.pone.0213282

**Published:** 2019-03-07

**Authors:** Yurika Ujiié, Katsunori Kimoto, Toyoho Ishimura

**Affiliations:** 1 Center for Advanced Marine Core Research, Kochi University, Nankoku, Japan; 2 Research and Development Center for Global Change, JAMSTEC, Yokosuka, Japan; 3 National Institute of Technology, Ibaraki College, Hitachinaka, Japan; Universitat Bremen, GERMANY

## Abstract

Marine protists play an important role in oceanic ecosystems and biogeochemical cycles. However, the difficulties in culturing pelagic protists indicate that their ecology and behavior remain poorly understood; phylogeographic studies based on single-cell genetic analyses have often shown that they are highly divergent at the biological species level, with variable geographic distributions. This indicates that their ecology could be complex. On the other hand, the biomineral (calcareous) shells of planktic foraminifers are widely used in geochemical analyses to estimate marine paleoenvironmental characteristics (i.e., temperature), because the shell chemical composition reflects ambient seawater conditions. Among the pelagic protists, planktic foraminifers are ideal study candidates to develop a combined approach of genetic, morphological, and geochemical methods, thus reflecting environmental and ecological characteristics. The present study precisely tested whether the DNA extraction process physically and chemically affects the shells of the planktic foraminifer *Globigerinoides ruber*. We used a nondestructive method for analyzing physical changes (micro-focus X-ray computed tomography (MXCT) scanning) to compare specimens at the pre- and post-DNA extraction stages. Our results demonstrate that DNA extraction has no significant effect on shell density and thickness. We measured stable carbon and oxygen isotopes on the shell of each individual in a negative control or one of two DNA-extracted groups and detected no significant differences in isotopic values among the three groups. Moreover, we evaluated isotopic variations at the biological species level with regard to their ecological characteristics such as depth habitat, life stages, and symbionts. Thus, our examination of the physiochemical effects on biomineral shells through DNA extraction shows that morphological and isotopic analyses of foraminifers can be combined with genetic analysis. These analytical methods are applicable to other shell-forming protists and microorganisms. In this study, we developed a powerful analytical tool for use in ecological and environmental studies of modern and past oceans.

## Introduction

Marine protists are the most abundant eukaryotes in the pelagic realm; recent field-based studies have unveiled their high diversity and abundance in the photic and deep layers of the world’s oceans [1,2]. Several techniques, including *in situ* imaging and metagenomic analysis, have been used to assess the biomass and variability of marine protists along horizontal and vertical dimensions of the oceans [3,4]. The results of previous studies suggest that protists greatly affect marine ecosystems [1–4]. In particular, protists represent the main component of the marine food web and act as a driving force for biogeochemical cycles [5]; therefore, knowledge of their ecology and environmental responses is important to understanding these roles. Although transcriptome analyses have been conducted to examine gene expression in protists from an ecological point of view [6], such metadata-based approaches (e.g., transcriptome and metagenomic analyses) have been limited to culturable species. Since most protists are difficult to culture, their ecology has remained unknown to date.

Single-cell sequencing techniques have been developed to obtain DNA sequences from unculturable protists and utilized to clarify phylogenetic relationships for unveiling cryptic diversity within groups of protists [7–9]. In particular, numerous molecular studies have revealed the presence of many genetically incompatible species (biological species) among planktic foraminifers and shown their species-specific distribution in the world’s oceans (compiled in [10]). Planktic foraminiferal species were originally defined based on their biomineral (calcareous) shell morphology. The shells are used as paleontological and paleoenvironmental study subjects, because they fossilize and well preserve in marine sediments. Although planktic foraminifers are generally distributed in latitudinal provinces, from the tropical to polar oceans [11], biological species have more precise geographic distributions [10]. These species diversity and distribution patterns imply that they have greater species-specific ecological variation than has been traditionally understood. For example, though the left/right coiling direction of the shells of *Neogloboquadrina pachyderma* and *Globorotalia truncatulinoides* were thought to be temperature dependent, molecular phylogenetic studies demonstrated a genetic basis for coil-morph in planktic foraminifers [12,13]. Moreover, population sampling of planktic foraminifers and closely related radiolarians has revealed unexpected dispersal patterns: first, the dispersal of planktic foraminiferal populations is not facilitated by ocean currents [14,15]; and, second, two sibling radiolarian species have been found to be distributed at different depths along the water column according to food resource availability [16]. These studies demonstrate that genetic differentiation of pelagic protists is associated with ecological differences among sibling species. Another study, which estimated the precise divergence time of planktic foraminiferal sibling species considering their phylogeography, has suggested that ecological adaptation to environmental changes could be a strong driving force in the diversification of pelagic protists, in the absence of effective physical barriers to gene flow [17]. However, although such single-cell investigative approaches have enhanced the recognition of ecological roles in the evolution of pelagic protists, their ecology cannot be fully understood based solely on genetic analyses. Therefore, we need to advance our knowledge of protistan ecology by utilizing methods that also analyze morphological and biogeochemical traits.

To consider multiple factors–genetic, morphological, and geochemical traits–in marine protistan ecology, biomineral shell-bearing protists, like planktic foraminifers, are good study candidates. The calcareous shells of planktic foraminifers have already been used in morphological and isotopic analyses. Stable carbon (*δ*^13^C) and oxygen (*δ*^18^O) isotopic compositions of shells are widely used as environmental proxies in paleoceanography, because the shells form under the influence of ambient seawater conditions [18]. Changes in the oceanic CO_2_ cycle with the atmosphere have been estimated for the Quaternary based on *δ*^13^C and *δ*^18^O changes derived from foraminiferal fossil shells [19,20]. On the other hand, planktic foraminiferal *δ*^13^C and *δ*^18^O values are affected by physiology and metabolism, i.e., vital effects [21–23]. In addition, many planktic foraminiferal species harbor algal symbionts [24]. Several experiments have revealed that the photosynthetic activity of these algal symbionts, which varies according both to increasing symbiont density with host-cell growth and differences in habitat light intensity, will greatly affect the planktic foraminiferal *δ*^13^C values [25–27]. The effect of photosynthesis on planktic foraminiferal *δ*^13^C and *δ*^18^O has been estimated based on comparison measurements between symbiont-bearing and -barren morphospecies [21,22]. However, these previous studies did not focus on the differences in vital effects at the biological species level (i.e., between genetic types). In this context, isotopic traits are integrated by both the ecology and physiology of biological species of both planktic foraminifers and symbionts. The genetic information from DNA samples could identify more valid ecological and environmental roles for marine protists than those currently known.

The morphology of planktic foraminiferal shells, which is useful to identify morphospecies, can advance novel tools in multiple fields. Ocean acidification physically alters the calcareous shells of planktic foraminifers, making them thinner and lighter [28], although existing morphological measurement techniques do not allow these changes to be accurately quantified in very small organisms. Development of a method for precise morphological measurement could elucidate slight changes in calcareous shells according to global changes. Moreover, past molecular studies have modified DNA extraction methods so that foraminiferal calcareous shells are kept intact [29]. These preserved shells have been used in morphometric analyses to reassess the morphological characteristics of biological species [30,31]. The combination of DNA sequences and shell morphology enable modern ecological information for each biological species to be extrapolated to fossil specimens; this is critical because it is impossible to obtain useful DNA sequences from the latter. In light of the high potential of planktic foraminiferal DNA and shells to provide novel information on ecological, evolutionary, and (paleo)environmental changes, new approaches are needed that utilize a combination of genetic, morphological and geochemical factors.

The currently used DNA extraction method comprises an incubation step at 65–70°C in a chemical buffer [32], but no study has considered that this experimental step would exert physical and chemical damage on shells. In the present study, we employed the micro-focus X-ray computed tomography (MXCT) scanning method for physical (i.e., density) and morphological analyses. As this is a non-destructive method, it allowed us to measure and observe the microscale surface and internal structures of the shells without damaging them, and to preserve the specimen for other experiments. In order to examine isotopic changes in shells, we determined the *δ*^13^C and *δ*^18^O values of each shell using a microscale isotopic analytical system. Through these experiments, we were able to test changes to physical/morphological and stable isotopes in planktic foraminiferal shells caused by the DNA extraction process and, moreover, develop a novel approach for integrating DNA, morphology, and stable isotope analyses for each individual protist.

## Materials and methods

### Materials

**Fig 1:**
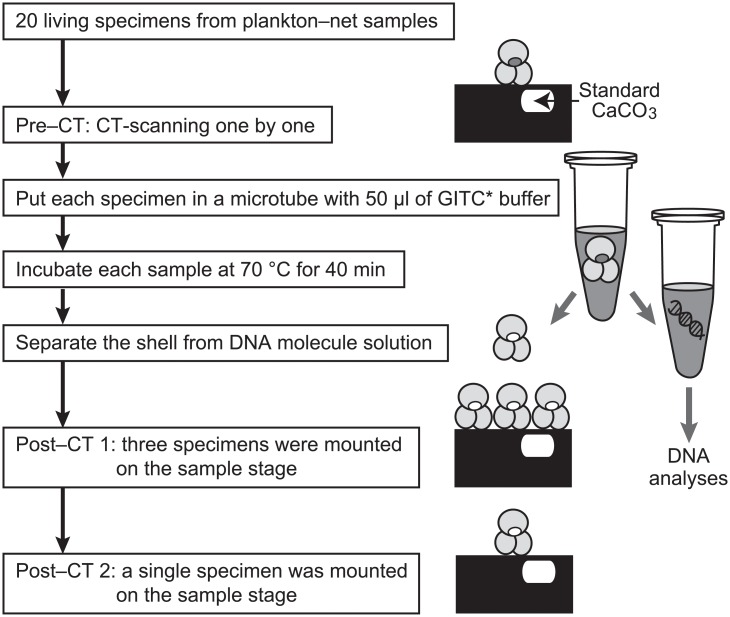
Analytical flowchart for the micro-focus X-ray computed tomography (MXCT) scanning method combined with genetic analyses. Pre- and Post-computed tomography (CT) show MXCT scanning before incubating shells in the DNA extraction buffer (modified guanidine isothiocyanate (GITC*) buffer) and after. Post-CT 1 and Post-CT 2 show different mounting of specimens on the sample stage.

**Fig 2:**
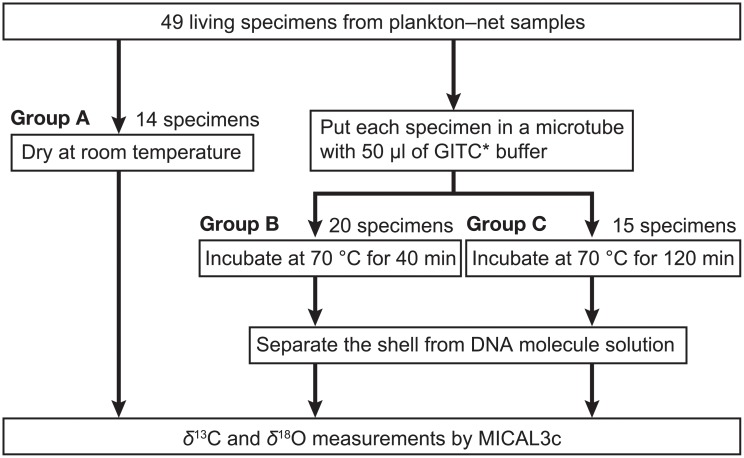
Analytical flowchart for *δ*^13^C and *δ*^18^O measurements combined with genetic analyses. We used a mass spectrometer for isotopic analysis of the shells (MICAL3c).

We collected plankton-net samples at two sites in the northwestern Pacific Ocean: one off Manazuru, Japan (35°10ʹN, 139°11ʹE, 602 m depth), and the other off Kochi, Japan (33°15ʹN, 133°38ʹ15E, 200 m depth). No specific permissions were required to conduct the sampling, and the study did not involve endangered or protected species. At both sites, a net (100 μm mesh) was vertically towed from above the chlorophyll maximum layer (~70 m water depth) to the surface. After net towing, we immediately collected living specimens of the planktic foraminifer *Globigerinoides ruber* from the bulk net-samples under a microscope. These specimens were larger than 100 μm and have identical morphology as *G*. *ruber*. In all the studied specimens, three globular chambers per whorl were observed on the umbilical side and small secondary apertures on the spiral side. Specimens with these morphological characters are categorized as the adult stage among five stages (prolocular, juvenile, neanic, adult, and terminal stages) [10]. This morphospecies, which has algal endosymbionts [24], is composed of five biological species [33]. Twenty specimens collected off Manazuru were rinsed with sterilized water, dried at room temperature (~22°C), and preserved for MXCT scanning analysis (Fig 1). Forty-nine specimens collected off Kochi were randomly divided into three groups: group A (14 specimens) received the same treatment as those collected off Manazuru; and groups B and C (20 and 15 specimens, respectively) were preserved in a DNA extraction buffer (Fig 2).

The DNA extraction buffer (GITC*) contains 8.5 M guanidinium isothiocyanate (GITC), 0.106 M Tris-HCl (pH 7.6), 4.24% sodium N-lauroyl-sarcosinate, and 2.1% β-mercaptoethanol in sterilized water [32]. GITC is commonly used for protein denaturation in DNA and RNA extraction from cells, such as bivalve mollusks [34], coral [35], and radiolarians [16]. GITC* was modified from the GITC protocol to preserve calcareous shells by removing ethylenediaminetetraacetic acid (EDTA) [29,32]. As a standard procedure, each specimen was incubated in 50 μl GITC* buffer at 70°C for 40 min. After this step, the shell was separated from the buffer that included the extracted DNA and rinsed by sterilized water to remove remaining buffer. Each of the 20 specimens collected off Manazuru was subjected to the same standard DNA extraction process after MXCT scanning analysis (Fig 1). For stable isotope analysis, each of the 20 specimens in group B was subjected to the standard DNA extraction process, while each of the 15 specimens in group C was incubated for 120 min (i.e., three times longer than the standard procedure) for DNA extraction (Fig 2).

### MXCT scanning analysis

We used a ScanXmate-D160TSS105 (Comscantecno Co. Ltd., Kanagawa, Japan) for MXCT scanning. This system applies X-rays to the sample stage, which can rotate through 360 degrees, with high-resolution settings (X-ray focus spot diameter, 0.8 μm; X-ray tube voltage, 80 kV; detector array size, 1024 × 1024; 1200 projections/360°, four-times averaging, sequential imaging, 2.0 s/projection). The recording time was ~40 min. Spatial resolution of transmitted image was 0.8 μm/pixel.

Three-dimensional (3D) tomography was reconstructed using the convolution back projection method contained in ConeCTexpress (Comscantecno Co. Ltd.). The physical structure of each planktic foraminiferal shell was calculated using Molcer Plus software (White Rabbit Corp., Tokyo, Japan).

The 16-bit grayscale contrasts (65,535 gray level gradations) of a transparent image achieved by MXCT indicate the degree of X-ray attenuation of an object. The grayscale contrasts reflect the relative density and/or relative atomic mass of the objects. However, these contrasts are not steady due to fluctuations in the X-ray beam energy spectrum during the scanning time and other imaging artifacts. Although it is difficult to clear all the artifacts that exist on an X-ray system, we successfully developed a novel analytical protocol to reduce the effects of X-ray beam energy spectrum fluctuations in the present study. We acquired transparent images of each object, together with standard calcite under the same conditions of X-ray beam energy, and normalized the grayscale contrast of the object to that of the standard material. This standardization mathematically canceled the X-ray spectrum fluctuations.

In medicine, the computed tomography (CT) number is widely used to show human bone density, and is calculated using the following equation:

$$CT\;number=k\times (\mu_{m}-\mu_{w})∕\mu_{w}$$

where *k* is a constant (1000), and μ_m_ and μ_w_ are the X-ray attenuation coefficients of the object and water, respectively. The CT number is expressed in Hounsfield units (HU) and represents the relative density of an object compared with the density of a calibration phantom, which are considered to be 0 and –1000 HU for water and air, respectively. In the present study, we modified the CT number equation to show the bulk density of the calcareous object (i.e., planktic foraminiferal shell); this calcite CT number (CCN) was calculated as:

$$CCN=1000\times \left[(\mu_{sample}-\mu_{air})∕(\mu_{calcite}-\mu_{air})\right]$$

where μ_sample_, μ_air_, and μ_calcite_ are the X-ray attenuation coefficients of the object, surrounding air, and standard calcite, respectively. Here, the highest density (2.71) of pure calcite crystal was set as 1000 in the CCN. A block of calcite crystal NIST NBS-19, which is the certified reference material for *δ*^13^C and *δ*^18^O in geochemical/earth science laboratories, was employed as the standard material. We selected the most homogeneous calcite grains from the available NIST NBS-19 grains to avoid calcite heterogeneity caused by small voids and contamination by other minerals. This standard calcite was embedded in urethane adhesive on the sample stage.

**Fig 3:**
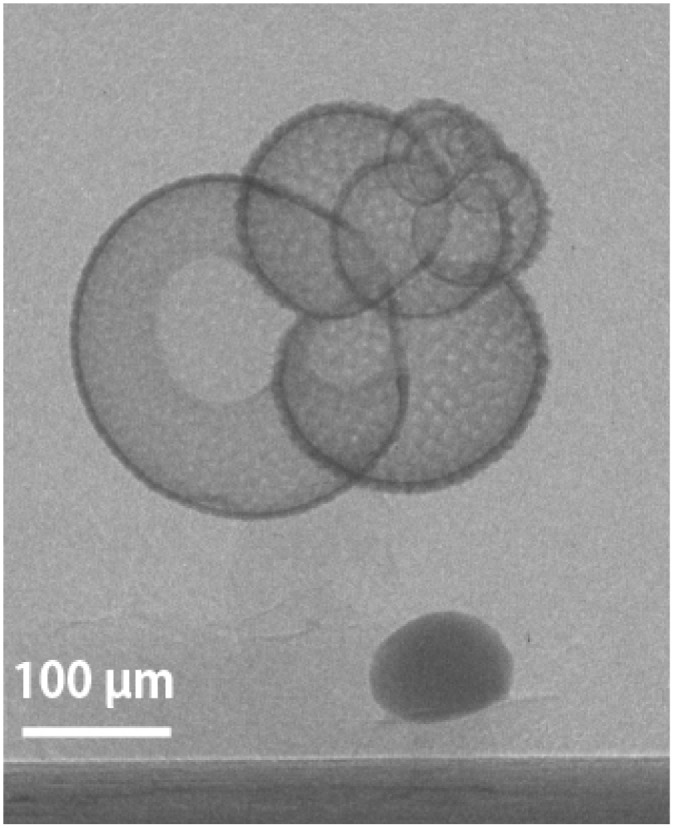
X-ray transparent image of a *Globigerinoides ruber* specimen (ID #1 in Table 1). The specimen is mounted on the sample stage. The dark-gray orbicular object under the specimen is standard calcite. The urethane adhesive is not visible in the X-ray transparent image. The black bar at the bottom is the sample stage.

Each of the 20 planktic foraminiferal specimens collected off Manazuru was mounted on the sample stage with urethane adhesive (Fig 3). All 20 specimens were scanned individually (pre-CT analysis), and then incubated in GITC* buffer. After separating the shell from the extraction buffer, each specimen was reanalyzed using the MXCT scanning system (post-CT analysis). Moreover we conducted post-CT analysis twice to test the effect of X-ray beam-hardening (see Results and discussion): first, by mounting groups of three specimens on the sample stage and scanning them together (post-CT 1); and, second, by mounting and scanning each specimen on the sample stage individually (post-CT 2) (Fig 1).

### Stable isotope analysis

The *δ*^13^C and *δ*^18^O values of the planktic foraminiferal shells were measured using an IsoPrime100 isotope ratio mass spectrometer with a customized continuous flow gas preparation system (MICAL3c; [36,37]). This system comprises a microvolume CaCO_3_ decomposition tube, stainless steel CO_2_ purification vacuum line with a quantity-regulating unit, helium-purged CO_2_ purification line, gas chromatograph, and a continuous-flow isotope ratio mass spectrometry system. The continuous flow method permits isotopic analysis of ~0.2 μg CaCO_3_. The external precision of this system is better than ± 0.10% for both *δ*^13^C and *δ*^18^O [37,38]. Moreover, in this system, the mass of calcite in foraminiferal shells is calculated from the volume of CO_2_ gas obtained during their reaction with phosphoric acid [36]. This high-precision analysis for small sample amounts has previously been applied to single specimens of planktic and benthic foraminifers [39,40], and to a dissected chamber of a planktic foraminiferal shell [41,42]. These studies demonstrate the existence of isotopic variations among individuals and growth stages. This analytical method is therefore suitable for comparisons of *δ*^13^C and *δ*^18^O among individuals.

The reaction of the calcareous shells with phosphoric acid to CO_2_ gas in the MICAL3c system makes it impossible to compare isotope values before and after DNA extraction on the same specimen, in *δ*^13^C and *δ*^18^O analyses. We used different DNA-extraction procedures to compare the *δ*^13^C and *δ*^18^O values in the 49 specimens from the three groups (groups A–C; see Materials section) (Fig 2). All data are reported in standard *δ* notations (*δ*^13^C and *δ*^18^O; ‰) relative to Vienna Pee Dee Belemnite (VPDB).

### Statistical analysis

A paired t-test was conducted to evaluate differences between the mean CCNs of two datasets (pre-CT and post-CT 2; or post-CT 1 and post-CT 2), because the same subject was measured during each of pre- and post-CT analyses with the MXCT scanning system. The null hypothesis (no differences between the means of paired datasets) could be accepted.

For the *δ*^13^C and *δ*^18^O data, the three groups (A–C) were compared. We firstly tested normality for each of the *δ*^13^C and *δ*^18^O datasets by Kolmogorov-Smirnov and Shapiro-Wilk tests. According to the results of this test, we analyzed differences among the three datasets by a parametric (one-way analysis of variance; ANOVA) and nonparametric (Kruskal–Wallis) tests. The null hypothesis (no differences in the isotopic ratio among the three groups) could be accepted.

### DNA amplification and phylogenetic analysis

Genomic DNA was extracted from each specimen in groups B and C, the shells of which were used for stable isotope analysis. Approximately 800 base pairs from the terminal 3ʹ end of the small subunit ribosomal DNA (SSU rDNA) were amplified by polymerase chain reaction (PCR) analysis, using primers s14p (5ʹ-AAG GGC ACC ACA AGM GCG-3ʹ) and sBf (5ʹ-TGA TCC ATC RGC AGG TTC ACC TAC-3ʹ) [7]. The following PCR conditions were maintained: 40 cycles of 95°C (30 s), 56°C (30 s), and 72°C (1 min 30 s), with a final elongation step of 10 min at 72°C. The PCR products were purified using ExoSAP-IT reagent (Affymetrix Inc., CA, USA), and sequenced with PCR primers using the ABI Prism 3130 Genetic Analyzer (Applied Biosystems, CA, USA) at the Center for Advanced Marine Core Research, Kochi University. Thirty-four SSU rDNA sequences were deposited in the GenBank (accession numbers LC413854–LC413887).

Fifty-three SSU rDNA nucleotide sequences, which included 19 published sequences from GenBank (their accession numbers are shown in S1 Fig), were manually aligned with the SeaView v4.3.4 program [43]. After excluding ambiguously aligned sites, 769 sites of SSU rDNA sequences were chosen for phylogenetic analysis. The Hasegawa–Yano–Kishino (HYK) model [44] with a gamma (Γ) [45] distribution for variable rates was selected as the best-fit nucleotide substitution model, using MrModelTest 2.3 [46]. Bayesian analyses were conducted on the SSU rDNA dataset with the optimal models, using the MrBayes v 3.2.6 program [47]. The Markov Chain Monte Carlo (MCMC) process was set to enable simultaneous functioning of four chains (three heated and one cold). Two independent runs were conducted for 1.2 × 10^6^ generations. The trees and log-likelihood values were sampled at 100-generation intervals. The first 2 × 10^5^ generations were excluded as burn-in. Pooled trees (1.0 × 10^6^ generations) were used to obtain the Bayesian posterior probabilities for each dataset. The maximum likelihood (ML) analysis for the same dataset was performed using Treefinder [48], and bootstrap support was based on 1000 replicates in each dataset.

## Results and discussion

### Physical changes in shells

First, we compared the CCNs of the post-CT 1 and post-CT 2 analyses, which measured the same specimens mounted on the sample stage either in groups of three or individually, respectively (Fig 1, Table 1). The CCNs of post-CT 1 and post-CT 2 analyses differed by approximately 20.8 on average; this difference was statistically significant (p < 0.05, paired t-test). This is caused by beam-hardening, in which the lower energy photons of a polychromatic X-ray beam passing through an object are easily absorbed, leaving the higher energy photons. When the X-ray beam is transmitted through three specimens, low energy X-rays are absorbed in the front objects. In the MXCT system, a metal filter (0.2-mm-thick aluminum plate) is set between the X-ray source and object to mask low energy X-rays and allow selective detection of higher energy X-rays. This filter largely reduces the beam-hardening effect of an object within a depth of 150 μm. However, mounting three specimens on the sample stage, as in the present study, could have exceeded that depth. The above-mentioned differences provided an incentive for making effective changes to the method used for MXCT scanning analysis of the microscale samples.

**Table 1:** Calcite computed tomography (CT) number (CCN) and shell thickness of the planktic foraminifer *Globigerinoides ruber*.

ID	Pre-CT		Post-CT 1		Post-CT 2		CCN difference	
	CCN	Thickness (μm)	CCN	Thickness (μm)	CCN	Thickness (μm)	post-CT1 and post-CT2	pre-CT and post-CT2
1	795.0	3.67	758.0	3.65	799.6	3.52	−41.6	−4.5
2	825.4	3.73	812.6	4.15	796.0	4.14	+16.6	+29.4
3	860.5	4.72	806.9	4.82	855.6	4.59	−48.7	+4.8
4	781.8	3.63	750.9	3.57	812.6	3.55	−61.6	−30.8
5	819.8	3.35	773.2	3.72	797.8	3.43	−24.5	+22.0
6	708.0	2.61	687.5	2.31	699.5	2.38	−11.9	+8.5
7	815.3	3.42	747.1	3.41	788.7	3.37	−41.6	+26.6
8	810.3	3.51	754.8	3.50	764.5	3.42	−9.8	+45.8
9	866.7	3.99	821.0	4.13	821.5	4.16	−0.5	+45.2
10	780.3	3.12	775.7	3.01	744.8	3.19	+30.8	+35.4
11	800.0	4.16	804.3	3.99	805.2	4.12	−0.9	−5.2
12	797.4	3.55	764.8	3.40	787.1	3.45	−22.3	+10.3
13	823.0	4.30	812.4	3.96	795.9	4.21	+16.5	+27.1
14	865.9	4.47	815.0	4.23	848.8	4.17	−33.8	+17.1
15	772.4	3.90	798.3	3.52	829.4	3.54	−31.1	−57.0
16	849.9	4.45	850.3	4.29	855.0	4.25	−4.7	−4.7
17	781.0	2.98	726.9	2.94	783.6	2.87	−56.7	−2.6
18	806.3	3.44	749.8	3.48	803.2	3.42	−53.3	+3.2
19	846.6	4.20	815.3	4.26	840.9	4.19	−25.7	+5.7
20	740.2	2.85	737.7	2.65	748.9	2.71	−11.2	−8.7

Pre-CT and post-CT results show the values before and after DNA extraction, respectively. Post-CT 1 and post-CT 2 values were acquired from analyzing three specimens together and individually, respectively. Differences in CCNs were calculated from post-CT1 to post-CT2 and from pre-CT to post-CT2, respectively.

In contrast, the CCNs of pre-CT and post-CT 2 analyses of the same specimen differed by only ~8.4 on average and presented a variation of 1.05% (Table 1). These differences were not statistically significant (p > 0.05, paired t-test). Compared with the general CT analysis method, in which the variation in the CT number is ~10% for a 600 mm phantom [49], the present method showed a much higher accuracy (~1.05%) for a target equal to a few hundred micrometers. Even using the same instrument, the differences in CCNs between pre-CT and post-CT 2 analyses were smaller than those between the two different scanning methods (i.e., post-CT 1 and 2). These results indicated that our method using MXCT is accurate for analyzing microscale samples, and that no changes in shell density occurred during DNA extraction. Moreover, in the pre-CT and post-CT 2 analyses, the calculated thickness of the shell differed by between −0.41 and +0.37 μm, with an average difference of 0.07 μm (p > 0.05, paired t-test) (Table 1). These data showed little change after DNA extraction, although planktic foraminiferal calcite shells are only ~4 μm thick. Thus, the calcite shells of planktic foraminifers showed no physical damage after incubation in the DNA extraction buffer (GITC*) at 70°C for 40 min.

**Fig 4:**
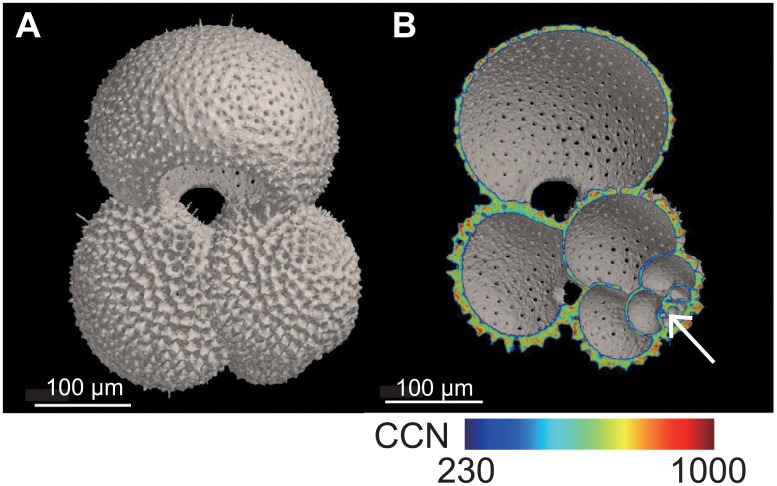
Three-dimensional tomograms of the shell of a *Globigerinoides ruber* specimen (ID #1 in Table 1). Tomogram of the exterior (4A). CT cross-section image of the same shell with coloring according to calcite density (4B). The white arrow marks the first chamber. Planktic foraminifers secrete calcite and grow chamber by chamber.

The 3D tomograms of the examined planktic foraminifers were reconstructed (Fig 4). We clearly observed the calcite wall ultrastructure, such as pores and spines in the exterior of the shell (Fig 4A), and ontogenetic structures from the first chamber in the CT cross-section image (Fig 4B). These 3D tomograms will be useful in precise morphometric analyses in order to define the morphological characteristics of biological species and their ontogeny, as MXCT is a powerful non-destructive method. In the previous studies, the exterior structures of planktic foraminiferal shells (e.g., pore distribution on the shell surface and external shell form) were used to define the morphological characteristics of genetic types [30,31]. These characters, however, were not sufficient to classify all presented genetic types [31]. The 3D tomograms provide more measurement points, to establish more detailed morphological characters, because they can use internal and ontogenetic structures in any direction. The ontogenetic structures could be helpful to distinguish morphological criteria for juvenile specimens and to understand the growth process [50,51]. In addition, this MXCT technique is non-destructive and applicable to other shell-bearing organisms: radiolarians, ostracods, small gastropods, and pteropods, as shown in pelagic gastropods [52]. Moreover, the calcite density distribution, which was based on CCNs, was visualized in color gradations (Fig 4B) at sufficiently high resolution for evaluating shell structure and the effects of anthropogenic ocean acidification and calcite dissolution [53,54]. The calcareous shells, including planktic foraminiferal shells, seem to dissolve because pH and carbonate ion concentration are reduced in the upper water column [55]. Regarding the acceleration of ocean acidification by increasing anthropogenic CO_2_ in the atmosphere, previous studies have tested the dissolving processes of the planktic foraminiferal shells in the field and the pH-controlled experiments by using MXCT scanning analyses [53,54]. One of these former studies reported that the shells dissolved from the inner chamber wall as initial dissolution, which was not observed from the external shell morphology [53]. The modification of scanning the standard calcite, with the samples as our MXCT analytical method, showed that shell density was reduced due to selective dissolution around the shell pores [54]. These physical changes in calcareous shells advanced our understanding about the effects of ocean acidification. Thus, MXCT scanning analysis of small organisms with biomineral shells could be applied in many types of studies, both in biological and paleontological fields.

### Isotopic changes in shells

**Fig 5:**
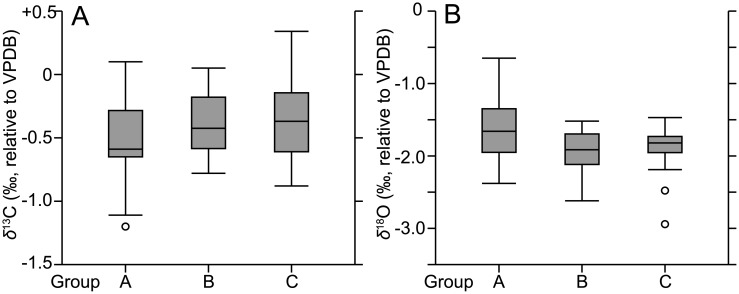
Box and whisker plots of *δ*^13^C and *δ*^18^O in three groups (A–C) of *Globigerinoides ruber*. Values for *δ*^13^C and *δ*^18^O are shown on the left (5A) and right (5B), respectively (i.e., n = 14 for group A, n = 20 for group B, and n = 15 for group C). Individual open circles correspond to outliers.

We obtained *δ*^13^C and *δ*^18^O values from each of the 49 specimens collected off Kochi (groups A–C; Table 2). The normality tests (Kolmogorov-Smirnov and Shapiro-Wilk) applied to these datasets revealed that the *δ*^13^C values in the three groups showed normal distributions, while *δ*^18^O values did not (Fig 5). Accordingly, the *δ*^13^C dataset was analyzed by a one-way ANOVA and the *δ*^18^O dataset by a Kruskal–Wallis test. In both datasets, there were no significant differences among the three groups (p > 0.05), even though the incubation time for DNA extraction was three times longer for group C than group B (120 min and 40 min, respectively). Our comparative experiments determined that the DNA extraction reagent has no effect on the *δ*^13^C and *δ*^18^O values of planktic foraminiferal shells. Previous diffusion experiments using calcite crystals have revealed that carbon and oxygen diffusion distances are 0.02 and 0.15 μm after heating at between 500 and 600°C for 3 h [56]. In our experiment, the incubation temperature was almost 10 times lower than the diffusion experiments. The diffusion distances of carbon and oxygen are negligibly small, even in thin (~4 μm) calcareous shells. Thus, our experiment showed that the incubation temperature (65–70°C) for DNA extraction also had no effect on the *δ*^13^C and *δ*^18^O values of planktic foraminiferal shells. We have therefore demonstrated it is possible to conduct stable isotopic analyses of planktic foraminiferal shells that have undergone a DNA extraction process, and that our method is applicable not just to the present study, but also to future research. Moreover, the same main compound (GITC) is used for other organisms with calcareous shells, as shown in Material and Methods. Thus, the same DNA extraction method may be applied to other organisms to measure stable isotopes.

**Table 2:** Results for genetic type, *δ*^13^C and *δ*^18^O values, and shell weight of the planktic foraminifer *Globigerinoides ruber* from stable isotope analysis.

	ID	Type	*δ*^13^C (‰)	*δ*^18^O (‰)	Shell wt. (μg)
A	11-D1		−0.22	−1.35	6.1
A	11-D2		+0.10	−1.43	8.2
A	11-D3		−0.49	−1.69	5.2
A	11-D4		−0.20	−1.17	4.2
A	11-D6		−0.62	−1.19	3.6
A	11-D7		−0.65	−2.08	3.0
A	11-D8		−0.60	−1.71	1.7
A	11-D9		−1.20	−0.65	1.4
A	11-D10		−1.11	−1.46	1.2
A	11-D15		−0.60	−2.28	1.1
A	11-D16		−0.29	−1.95	1.5
A	11-D20		−0.92	−2.38	4.1
A	11-D21		−0.58	−1.63	3.9
A	11-D22		−0.35	−1.80	2.4
B	12–2	Ia	−0.40	−1.74	6.1
B	12–3	Ia	−0.19	−1.61	4.9
B	12–4	Ia	−0.52	−2.12	3.0
B	12–5	Ia	−0.12	−1.91	5.3
B	12–11	IIa	−0.62	−1.92	2.4
B	12–12	IIa	+0.05	−1.64	4.7
B	12–13	IIa	+0.00	−1.65	3.8
B	12–15	Ib	−0.59	−2.12	2.6
B	12–16	IIa	−0.11	−1.83	3.9
B	12–17	IIa	−0.29	−1.52	6.7
B	12–18		−0.17	−2.62	4.1
B	12–19	Ib	−0.52	−1.99	1.4
B	12–20	Ib	−0.47	−2.36	3.0
B	12–21	Ib	−0.35	−2.25	1.8
B	12–22	Ia	−0.58	−2.10	2.4
B	12–23	Ia	−0.45	−2.08	1.7
B	12–36	Ib	−0.78	−1.88	1.5
B	12–39	IIa	−0.72	−2.16	1.5
B	12–40	Ib	−0.77	−1.83	1.4
B	12–41	Ia	−0.39	−1.60	1.4
C	12–6	Ia	−0.43	−2.01	3.8
C	12–7	IIa	+0.13	−1.56	7.6
C	12–8	IIa	−0.05	−1.76	6.1
C	12–9	IIa	+0.34	−1.47	6.7
C	12–10	IIa	−0.24	−2.48	5.3
C	12–24	Ia	−0.45	−1.90	2.0
C	12–25	Ia	−0.32	−1.70	1.7
C	12–26	Ia	−0.88	−1.76	1.8
C	12–28	Ib	−0.37	−1.82	2.7
C	12–30	Ia	−0.70	−1.48	2.0
C	12–32	Ib	−0.52	−1.89	1.4
C	12–33	IIa	+0.09	−2.94	5.5
C	12–34	Ia	−0.32	−2.19	2.9
C	12–35	Ia	−0.75	−1.82	1.5
C	12–36	Ia	−0.72	−1.86	1.5

Groups (A–C) correspond to different sample treatments before isotopic measurements were made. DNA extraction was not conducted for specimens in group A. *δ*^13^C and *δ*^18^O values: (‰ Vienna Pee Dee Belemnite; VPDB). Shell weight was calculated from the volume of CO_2_ gas obtained during their reaction with phosphoric acid.

**Fig 6:**
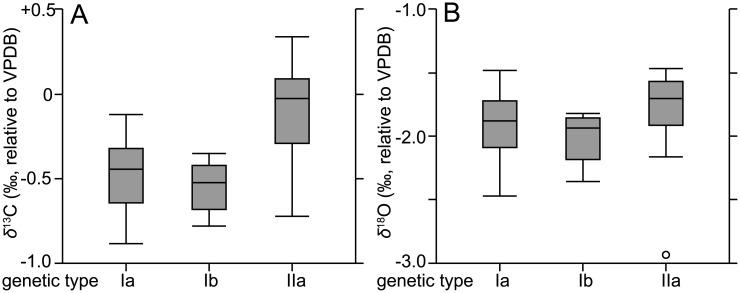
ANOVA results of *δ*^13^C and *δ*^18^O in three genetic types (Ia, Ib, and IIa) of *Globigerinoides ruber*. Values for *δ*^13^C and *δ*^18^O are shown on the left (6A) and right (6B), respectively (i.e., n = 16 for genetic type Ia, n = 8 for genetic type Ib, and n = 10 for genetic type IIa). Individual open circles correspond to outliers.

When *δ*^13^C and *δ*^18^O measurements in a single foraminiferal specimen were conducted, the previous studies often observed isotopic disequilibrium between shells and ambient seawater per species, presence/absence of symbionts, shell size, and calcification depth in the water column [21,22,27,40,57]. Some species show small variations in *δ*^13^C and *δ*^18^O that are almost in equilibrium with those observed in the surrounding water, whereas others do not [40]. Symbiont-bearing species show a large isotopic disequilibrium when compared with symbiont-barren species [21,22,57]. Because planktic foraminifers form chambers at different water depths during growth, their *δ*^13^C and *δ*^18^O values are associated with variations in the ambient seawater [41,42,57]. Planktic foraminiferal *δ*^13^C could be changed along the gradient of dissolved inorganic carbon (DIC) *δ*^13^C, if their calcification depths differ (deeper or shallower) at the ontogenetic stages. Our isotope data showed variability (Fig 5A), despite all examined specimens being collected by vertical towing the upper ~70 m at a shallow-water site (200 m depth to the bottom) and having symbionts. In such a shallow water depth, DIC is not largely changed (~0.3 ‰ the upper 100 m depth in the north Pacific Ocean) [58]. Therefore, isotopic differences in the present study were not caused by the presence or absence of symbionts and not strongly related to the DIC gradient in the water column. The previous studies reported that *δ*^13^C values of some species, including *G*. *ruber*, varied according to increases in foraminiferal shell size due to changing biological or kinetic fractionation effects, which are associated with abundance of symbionts in the cell or differences in metabolic activity between juvenile (small) and adult (large) specimens [26,27,57,59]. These effects seem to be species-specific [57,59]. Here, we investigated differences in *δ*^13^C values among biological species. Thirty-four out of 35 specimens within groups B and C were successfully sequenced and classified into three (Ia, Ib, and IIa) of the five known genetic types [33] (Table 2), based on the molecular phylogeny of partial SSU rDNA sequences (S1 Fig). Genetic types I and II are phylogenetically distant from each other, representing different biological species. By grouping genetic types, we were able to analyze variances in *δ*^13^C and *δ*^18^O values after normality tests. The *δ*^13^C values significantly differed among the three genetic types (p < 0.05, one-way ANOVA); in particular, the *δ*^13^C of genetic type IIa was statistically different from that of the other types (Fig 6A). In contrast, *δ*^18^O values were not significantly different among the three genetic types (p > 0.05, Kruskal–Wallis test) (Fig 6B). The *δ*^13^C variations suggested there were different vital effects among the foraminiferal biological species, probably associated with the ecological and physiological traits of each species.

**Fig 7:**
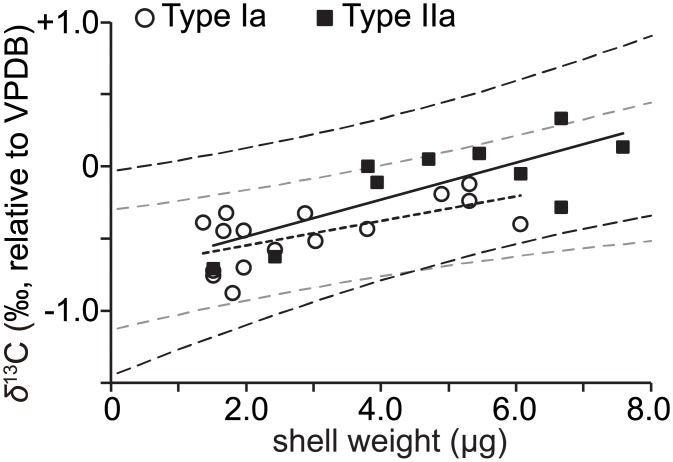
Changes in *δ*^13^C values in relation to shell weight in genetic types Ia and IIa of *Globigerinoides ruber*. Open circles correspond to type Ia and black squares to type IIa. Solid and dotted lines are the regression lines of types Ia and IIa, respectively. Black and gray dashed lines denote 95% confidence intervals for types Ia and IIa, respectively.

The correlations between the *δ*^13^C of *G*. *ruber* genetic types and the weight of calcareous shells, calculated as the volume of CO_2_ gas in the MICAL3c system, were examined. The weight of calcareous shells represents shell size, which was the sum of the length and breadth at the umbilical side of the shell measured by a digital microscope (VHX-2000, KEYENCE, Osaka, Japan) (S2 Fig). The shells of genetic type Ib, which ranged from 1.4 to 3.0 μg in weight (~350 to ~500 μm in length and breadth), varied little, compared with the other two genetic types (genetic type Ia: 1.4 to 6.1 μg in weight, 340 to 605 μm in length and breadth; genetic type IIa: 1.5 to 7.6 μg in weight, 320 to 660 μm in length and breadth) (Table 2, S2 Fig). Because it is not appropriate to test correlation within a small range, we examined the data on genetic types Ia and IIa, both of which ranged wide in shell weight. The *δ*^13^C of *G*. *ruber* genetic types Ia and IIa was positively correlated with the weight of calcareous shells (genetic type Ia: R^2^ = 0.643; genetic type IIa: R^2^ = 0.755) (Fig 7). More precisely, the regression line between *δ*^13^C and shell weight was steeper for type IIa (slope: 0.1281) than type Ia (slope: 0.0853); however, all data ranged within the 95% confidence interval for genetic type IIa (Fig 7). Although shell weight (size) of the studied specimens were in the same range between genetic types Ia and IIa (S2 Fig), probably indicating same ontogenetic stage, the *δ*^13^C values of genetic type IIa differed from the others (Fig 6A). Thus, the *δ*^13^C of genetic types changed according to shell weight (size), but their fluctuating breadths seem to differ between genetic types. In other planktic foraminiferal species, which have endosymbionts (dinoflagellates) like *G*. *ruber*, the *δ*^13^C values of *Globigerinoides sacculifer* and *Orbulina universa* increased, and they were associated with an increase in symbiont density during cell growth [25–27]. In the present study, we assumed that differences in the density and/or photosynthetic activity of symbionts between biological species (i.e., genetic types) affected *δ*^13^C values. If genetic type IIa is distributed slightly deeper than genetic type Ia along the water column, as has been observed in a previous field-based study [60], then these two genetic types are subject to different light conditions, which could result in different photosynthetic activities. These activities generate differences in the *δ*^13^C values of symbiont-bearing species [41]. However, in the present study, it was difficult to determine the effect of organismal depth distributions on *δ*^13^C because all specimens were collected by vertical towing at a shallow-water site (200 m depth). Another explanation for the differences found in *δ*^13^C values is that algal symbionts differ between planktic foraminiferal biological species and/or have different physiological traits. For example, two of the *Globigerinella siphonifera* genetic types were shown to host different algal symbiont types [8,61,62]. The light-absorption efficiency of the photosynthetic system differs between *G*. *siphonifera* and *G*. *sacculifer* [63], probably due to the specific adaptation of each algal symbiont to its host habitat. However, none of the previous studies has assessed isotopic differences between foraminiferal genetic types by considering the ribotypes of their algal symbionts. We will need to examine the algal symbiont characteristics in order to assess the vital effects on the *δ*^13^C and *δ*^18^O values of biomineral shells. These outcomes may reveal the biological effects on stable isotopes according to metabolic and physiological characteristics, both of planktic foraminifers and symbionts. They also highlight the environmental effects on the stable isotopes of foraminiferal shells in each biological species, which have specific geographic distributions. This will help to provide more accurate estimates of (paleo)environmental changes in the pelagic realm. Our method of combining genetic and isotopic analyses makes substantial progress toward understanding ecological and biogeochemical differences among planktic foraminifers, therefore providing important information for future studies of pelagic protists.

## Conclusions

In the present study, we made a thorough examination of the physiochemical effects of the processes used for DNA extraction in biomineral shells. Our objective was to establish a comprehensive method of analysis that integrates genetic, physical/morphological, and isotopic information for small, calcified microorganisms, such as planktic foraminifers. We developed a nondestructive analysis method, involving MXCT scanning, that was successful in obtaining accurate physical data (i.e., calcite density) and morphological images of the shells. Our results also showed that the DNA extraction process did not cause any physical changes in the shells. A microscale isotopic analytical system was used to measure the *δ*^13^C and *δ*^18^O values for single specimens. We designed a controlled experiment based on two groups, each of which experienced a different DNA extraction incubation time at 70°C, and a negative control group that did not undergo DNA extraction procedures. No significant differences in *δ*^13^C and *δ*^18^O values were observed among the three experimental groups, therefore demonstrating that the DNA extraction process did not cause isotopic changes in foraminiferal shells. The shell weight-*δ*^13^C regression slopes between the various genetic types of planktic foraminifers tend to differ from each other, and this is probably influenced by the ecological traits of biological species such as differences in the associated algal symbionts. These data could contribute to a better understanding of the vital effects on the stable isotopes of foraminifers and help us to more accurately reconstruct the environmental changes reflected in stable isotopes. Our study presents a powerful analytical tool for use with microscale samples. The physical/morphological and isotopic indicators complement the ecological aspects of unculturable pelagic protists and shell-forming microorganisms. These advances would support prospective studies of ecology and evolution in conditions of drastically changing pelagic environments.

## Supporting information

S1 Fig Phylogenetic reconstruction (Bayesian analysis, 50% majority consensus tree) based on partial small subunit ribosomal DNA sequences (769 base pairs) derived from individual *Globigerinoides ruber* specimens. Sequences obtained in the present study are shown in bold. Open and solid columns indicate clades of the five genetic types. Numbers at each node show posterior probabilities and bootstrap values.
(PDF)

S2 Fig Changes in the sum of the length and breadth at the umbilical side of the shell in relation to shell weight in genetic types Ia, Ib, and IIa. Open circles correspond to type Ia, gray triangle to type Ib, and black squares to type IIa. Black solid, gray solid, and dashed lines are the regression lines of types Ia, Ib, and IIa, respectively.
(PDF)
