# Supplementary figures and images for: Advanced approach to analyzing calcareous protists for present and past pelagic ecology: Comprehensive analysis of 3D-morphology, stable isotopes, and genes of planktic foraminifers
Source: PLoS One. 2019 Mar 7;14(3):e0213282. doi: 10.1371/journal.pone.0213282 (PMC6405064; doi:10.1371/journal.pone.0213282)

Supplementary Fig. S1\_Ujiie et al.

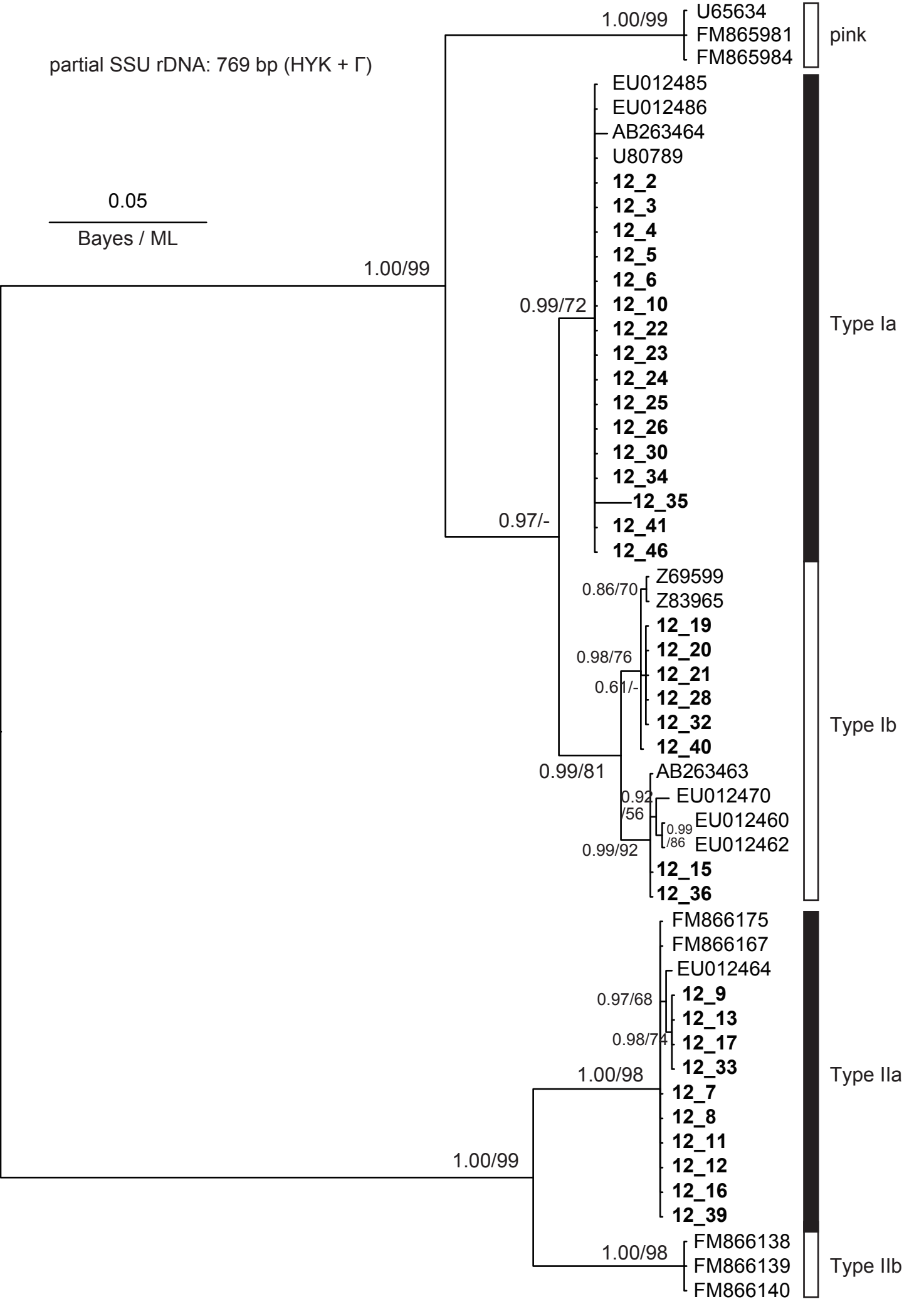

Supplement: S1 Fig — Sequences obtained in the present study are shown in bold. Open and solid columns indicate clades of the five genetic types. Numbers at each node show posterior probabilities and bootstrap values. (PDF) [file pone.0213282.s001.pdf]

Supplementary Fig. S2\_Ujiie et al.

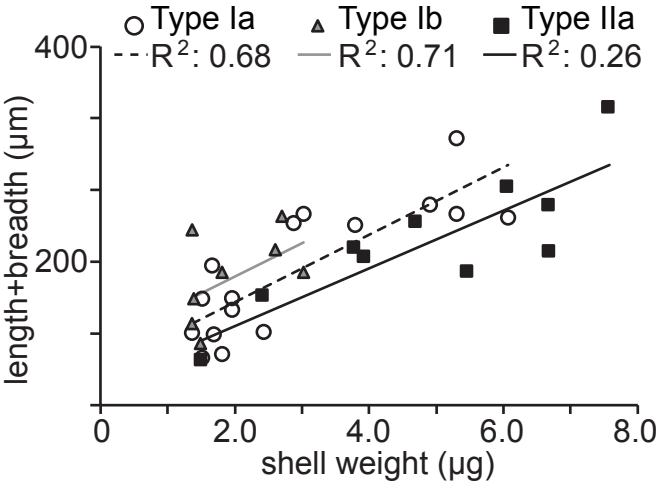

Supplement: S2 Fig — Open circles correspond to type Ia, gray triangle to type Ib, and black squares to type IIa. Black solid, gray solid, and dashed lines are the regression lines of types Ia, Ib, and IIa, respectively. (PDF) [file pone.0213282.s002.pdf]
